# Trends in Antimicrobial Susceptibility of *Escherichia coli* Isolates in a Taiwanese Child Cohort with Urinary Tract Infections between 2004 and 2018

**DOI:** 10.3390/antibiotics9080501

**Published:** 2020-08-10

**Authors:** Hung-En Chen, You-Lin Tain, Hsiao-Ching Kuo, Chien-Ning Hsu

**Affiliations:** 1 Division of Pediatric Nephrology, Department of Pediatrics, Kaohsiung Chang Gung Memorial Hospital, Chang Gung University, Kaohsiung 833, Taiwan; ten22022@cgmh.org.tw (H.-E.C.); tainyl@cgmh.org.tw (Y.-L.T.); 2 Department of Pharmacy, Kaohsiung Chang Gung Memorial Hospital, Kaohsiung 833, Taiwan; jeankuo@cgmh.org.tw; 3 School of Pharmacy, Kaohsiung Medical University, Kaohsiung 807, Taiwan

**Keywords:** *Escherichia coli*, urinary tract infection, community, children, antibiotics, resistance, antibiotic resistance, Taiwan

## Abstract

The aim of this study was to investigate the annual incidence of *Escherichia coli* isolates in urinary tract infections (UTIs) and the antimicrobial resistance of the third-generation cephalosporin (3GCs) to *E. coli*, including the factors associated with the resistance in hospitalized children in Taiwan. A large electronic database of medical records combining hospital admission and microbiological data during 2004–2018 was used to study childhood UTIs in Taiwan. Annual incidence rate ratios (IRR) of *E. coli* in children with UTIs and its resistant rate to the 3GCs and other antibiotics were estimated by linear Poisson regression. Factors associated with *E. coli* resistance to 3GCs were assessed through multivariable logistic regression analysis. *E. coli* UTIs occurred in 10,756 unique individuals among 41,879 hospitalized children, with 92.58% being community associated based on urine culture results reported within four days after the hospitalization. The overall IRR *E. coli* UTI was 1.01 (95% confidence interval (CI) 0.99–1.02) in community-associated (CA) and 0.96 (0.90–1.02) in healthcare-associated infections. The trend in 3GCs against *E. coli* increased (IRR 1.18, 95% CI 1.13–1.24) over time in CA-UTIs. Complex chronic disease (adjusted odds ratio (aOR), 2.04; 95% CI, 1.47–2.83) and antibiotics therapy ≤ 3 months prior (aOR, 1.49; 95% CI, 1.15–1.94) were associated with increased risk of 3GCs resistance to *E. coli*. The study results suggested little or no change in the trend of *E. coli* UTIs in Taiwanese youths over the past 15 years. Nevertheless, the increase in 3GCs-resistant *E. coli* was substantial. Interventions for children with complex chronic comorbidities and prior antibiotic treatment could be effective in reducing the incidence of 3GCs-resistant *E. coli* in CA-UTIs in this region and more generally.

## 1. Introduction

Urinary tract infection (UTI) is one of the most common severe bacterial infections in children, and more than 10% of children subsequently develop long-term complications, such as renal scars [1]. *Escherichia coli* of the *Enterobacteriaceae* family is the most common uropathogen in urinary tract infections (UTIs) [2,3], and the rise of antibiotic-resistant *E. coli* in pediatric UTI populations is a cause for concern in Taiwan [4,5] and around the whole world [6,7]. *E. coli* with resistance to third-generation cephalosporins (3GCs) is a suitable surrogate marker for identifying extended spectrum beta-lactamase (ESBL) and AmpC beta-lactamases producing organisms [8]. Furthermore, 3GC resistant *E. coli* isolates exhibit co-resistance to other classes of antibiotics, recurrent UTI, and require extended hospital stays in children with community-associated UTIs (CA-UTIs) [9,10,11].

Recent data suggest that the prevalence of 3GCs-resistant *E. coli* isolates in CA-UTIs has increased over time in adult populations [12,13]. Few studies have assessed longitudinal trends in pattern of antimicrobials resistance in childhood UTIs at a population level. Although prior studies have focused on antibiotic resistant *E. coli* in children with UTIs, most were derived from single centers or case-control studies designed in various geographic regions [11,14,15,16,17,18,19,20]. Understanding the trends of *E. coli* antibiotic resistance over time can help to evaluate and revise the empirical antibiotic therapy guidelines for childhood CA-UTIs at a country and regional levels.

The objective of this study was to assess the annual incidence of *E. coli* UTIs and antimicrobial susceptibilities of *E. coli* isolates between 2004 and 2018 in a large multicenter hospitalized children cohort in Taiwan. We hypothesized that both temporal trends in *E. coli* isolates in childhood UTIs and 3GCs-resistant *E. coli* differ over the 15-year period. In addition to the 3GCs-resistance rate, the present study was to explore the factors associated with 3GCs-resistant *E. coli* in children with CA-UTIs.

## 2. Materials and Methods

### 2.1. Study Design and Data Source

In this observational study, we used a large electronic database of health records on *E. coli* CA-UTIs in Taiwanese youth under 18 years of age. The study employed the Chang Gung Research Database (CGRD), which is derived from a group of Chang Gung Memorial Hospitals (CGMHs), an integrated healthcare system of 7 hospitals spanning Northern to Southern Taiwan. CGMHs comprise approximately 10–12% of the healthcare services covered by the Taiwan National Health Insurance (NHI) program [21]. The Taiwan NHI program is a compulsory single-payer health insurance program that covers over 99% of Taiwan’s population [22]. The CGRD contains detailed diagnostic data, prescription history, procedures, and laboratory test results from the emergency department (ED), as well as inpatient and outpatient settings.

**Figure 1:**
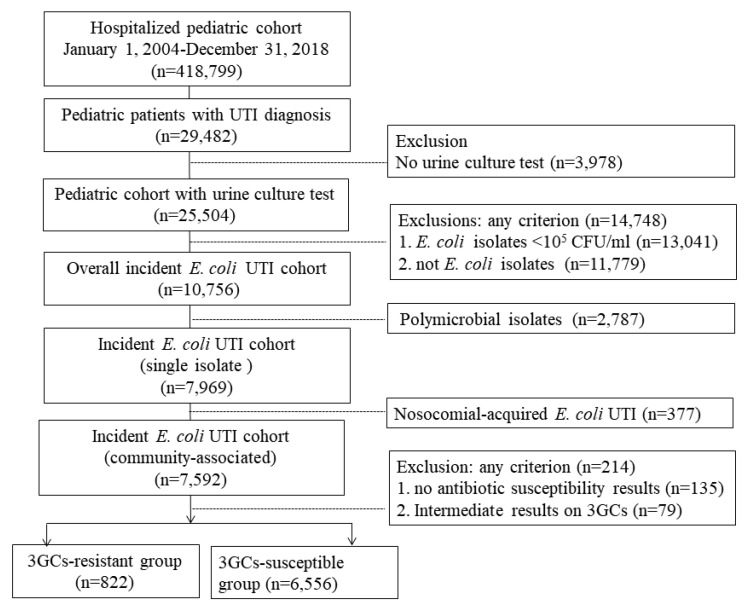
Study analysis flow of third-generation cephalosporins (3GCs).

We included all available data on *E. coli* UTIs between January 1, 2004, and December 31, 2018, including all bacteria isolated from urine cultures. Pediatric hospital admission was defined as patients aged 18 years or younger (Figure 1). The study proposal was approved by the Institutional Review Board of the Chang Gung Medical Foundation at Taipei, Taiwan (permit number: 201900903B0). The IRB approves the waiver of the informed consent form.

### 2.2. Outcome Measurements

The primary study outcome was the annual incidence of *E. coli* isolates in CA-UTIs among hospitalized children. Following current guidelines and the previous literature for community UTIs [12,16], an episode of UTI was defined as the presence of the following: (1) a discharge diagnosis of UTI, using the *International Statistical Classification of Diseases and Related Health Problems* codes (2004–2016: *Ninth Revision (ICD-9);* 2017–2018: *Tenth Revision* (*ICD*-*10*)) (Supplementary Materials Table S1) [10] and (2) a urine culture having more than 1 × 10^5^ colony-forming units (CFU) per mL of bacteria with a single uropathogen isolated to ascertain the associated burden [23]. To avoid over counting by including prior infections (e.g., recurrent UTI), only the first hospitalization (including transferred from emergency department and outpatient visit) for a UTI during the study duration was included (as the index hospitalization). Because antibiotic susceptibilities generally take approximately 3 days to report the resistant bacteria in the study setting, CA-UTIs were defined by the earliest urine culture specimen result reported within 4 days after the admission date of index hospitalization (approximately, urine samples collected within first 24–48 h after hospitalization). Children having a sample of urine culture during the index hospitalization (>4 days after the admission date or ≤3 days after discharge date) were considered to have healthcare-associated UTIs [24].

Secondary outcomes were *E. coli* resistance rates to the third-generation cephalosporins (3GCs: ceftriaxone or ceftazidime), as well as commonly recommended antibiotic regimens [22,23], including sulfamethoxazole–trimethoprim (SMT), gentamicin, ciprofloxacin, and cefazolin. The antimicrobial susceptibility testing results (R, resistant; S, susceptible; and I, intermediate) for the earliest *E. coli*-positive cultures were used to identify a case of *E. coli* resistant isolate in response to the specific antimicrobial agent. The individual antimicrobial resistant rate was calculated based on the number of children with CA-UTIs having an antimicrobial susceptibility test. Additional outcomes were healthcare service usage and in-hospital mortality. To investigate the impacts of patient characteristics and complexity of comorbid conditions on the group of 3GCs-resistant participants, the Pediatric Medical Complexity Algorithm (PMCA) [25,26] was employed and compared between sensitive and resistant groups (Supplementary Materials Table S1). To understand the contribution of an *E. coli* bloodstream infection around the time of an episode of *E. coli* CA-UTI, we investigated the discharge diagnosis of bacterial bloodstream infections from the index hospitalization.

### 2.3. Statistical Analysis

We identified incident cases of *E. coli* CA-UTIs among youths admitted for UTI which had a >10^5^ CFU/mL of any uropathogen in each calendar year during the study period. The trend of the annual incidence rate ratios (IRRs) with 95% confidence intervals (CIs) over time were estimated by using linear Poisson regression for *E. coli* in CA-UTIs and antibiotic resistance. In the Poisson regression, *E. coli* CA-UTIs were taken as the outcome, year as the predictor, and children having a clinically meaningful uropathogen were considered at risk, as an offset.

Furthermore, to compare the differences between single and mixed *E. coli* isolates in CA-UTIs, we also analyzed changes in the trend in IRR of *E. coli* CA-UTIs and the pattern of antibiotic susceptibility in *E. coli* with other uropathogenic isolates (with or without >10^5^ CFU/mL). Factors associated with 3GCs-resistant *E. coli* in children with CA-UTIs were determined by using adjusted odds ratios (aOR) with 95% confidence interval in multivariable logistic regression. All reported *p*-values were 2-tailed, and *p* < 0.05 indicated statistical significance. Data were analyzed by using SAS (Statistical Analysis System) version 9.4 (SAS Institute, Cary, NC, USA).

## 3. Results

From 2004 to 2018, there were 25,504 children with urine culture testing for *E. coli* UTIs among 418,799 hospitalized youths, with 10,756 of those patients having their first hospitalization for urine-culture-confirmed *E. coli*–isolated UTIs. Of patients with *E. coli* as a single uropathogen (*n* = 7969), 95% were community associated (Figure 1). On average, 1700 unique children received urine culture testing to confirm a UTI infection annually. Details regarding the annual number of hospital admissions combining isolated *E. coli* uropathogen results >10^5^ CFU/mL are presented in Supplementary Materials Table S2. The other most frequent UTI causative pathogens included *Enterococcus* species, following by *Klebsiella pneumoniae* and *Proteus mirabilis*, in the present study cohort.

Children < 1 year of age comprised approximately 80% of the incident *E. coli* CA-UTI cohort, with the mean age at the index hospitalization being younger in community cases than those deemed healthcare-associated (1.22 (±2.6) vs. 2.42 (±4.57), respectively; *p* < 0.0001). Pediatric patients with nosocomial UTIs were more commonly neonates (≤28 days) or youths in the 3–17-year age group, or those with complex chronic diseases. Characteristics of patients with community- or healthcare-related *E. coli* UTIs (both with single uropathogen) are presented in Supplementary Materials Table S3.

### 3.1. Incidence of E. coli in Community-Associated UTIs

**Figure 2:**
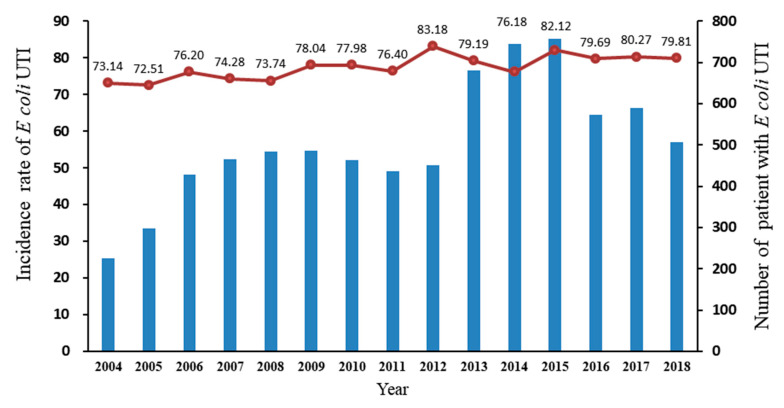
Incidence rate of community-associated *E. coli* (single isolate) UTI among children aged <18 years, 2004–2018 (IRR1.01, 95%CI (0.99–1.02), *p* = 0.2870). The red line indicates annual incidence rate of *E. coli* UTI, and the blue bars indicate the number of patients with *E. coli* UTI. The incidence rate ratio (IRR) was derived from a Poisson regression model based on the observed rate in each calendar year. The denominator was all patients with > 10^5^ CFU/mL any bacteria isolated UTI in each calendar year.

Figure 2 illustrates the number and annual incidence of *E. coli* isolates in CA-UTIs during the study duration. The overall incidence of CA-UTIs with *E. coli* single uropathogen did not significantly change between years (IRR 1.01, 95% CI 0.99–1.02, *p* = 0.2870), as well as *E. coli* isolated in healthcare-associated infections (IRR 0.96, 95%CI 0.90–1.02, *p* = 0.1724). The mean number per year was 728 unique children with *E. coli* CA-UTI between 2013 and 2015, which is higher than 416 per year between 2004 and 2012 and 557 per year between 2016 and 2018. The mean number per year over the full study duration was 506 unique children (2004–2018).

When considering UTIs with polymicrobial uropathogens, the overall incidence of *E. coli* uropathogen in CA-UTIs (single and polymicrobial) remained steady (IRR 1.01, 95% CI 0.99–1.02, *p* = 0.3855) over time (Supplementary Materials Figure S1). The mean number per year was 676 unique children with *E. coli* CA-UTIs from 2004 to 2018. The most common uropathogen found in mixed cultures was *Enterococcus faecalis*, followed by *Klebsiella pneumoniae* and *Proteus mirabilis*, without limitations to the colony counts ≥ 10^5^ UFC/mL.

### 3.2. Trends in Antimicrobial-Drugs-Resistant E. coli Isolates in CA-UTIs

**Figure 3:**
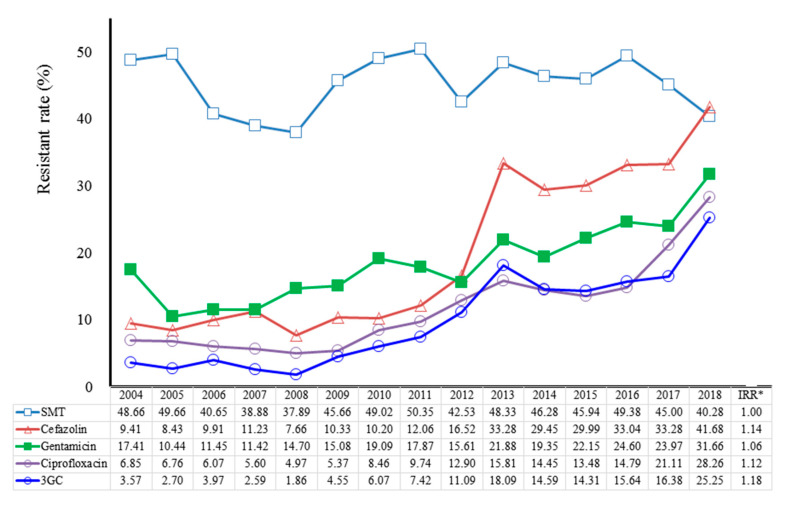
Trends of antibiotic resistance of community-associated *E. coli* UTI (single isolate) among children aged <18 years, 2004–2018. SMT, sulfamethoxazole–trimethoprim; 3GCs, third-generation cephalosporins. IRR*, incidence rate ratio, all *p*-values < 0.0001, except SMT (*p* = 0.9342). The mean IRR was derived from a Poisson regression analysis based on the observed rate in each calendar year. The denominator was the patients with *E. coli*–isolated UTI having the particular antibiotic susceptibility results across the study period.

Figure 3 illustrates the annual rate and trend changes in *E. coli* CA-UTI antibiotic resistance during the duration of the study. The incidence of resistance to 3GCs in *E. coli* CA-UTIs increased over the study duration, and over 10% per year since 2012 (2012–2018:11.09–25.25%). In addition, the risk of resistance to 3GCs was higher than other antibiotic regimens (IRR 1.18, 95% CI 1.13–1.23, *p* < 0.0001). An increasing trend (2012–2018: 12.9–28.26%) was observed for ciprofloxacin-resistant *E. coli,* which was broadly similar to 3GCs-resistnance in the CA-UTIs cohort (IRR 1.12, 95% CI 1.08–1.17, *p* < 0.0001). Although cefazolin- (IRR 1.14, 95%CI 1.11–1.17, *p* < 0.0001) and gentamicin-resistant *E. coli* (IRR 1.06, 95% CI 1.03–1.09, *p* < 0.0001) increased throughout the study period, 73.3% and 85.0% of 3GCs-susceptible *E. coli* isolates in CA-UTIs were susceptible cefazolin and gentamicin, respectively. Of gentamicin-resistant *E. coli* isolates (*n* = 1429), 99% were susceptible to amikacin. Antibiotic-resistant *E. coli* isolates to SMT was high (2004–2018: 48.66 to 40.28%), and the rates were sustained over time (IRR 1.01, 95% CI 0.99–1.02, *p* = 0.9342). Few patients (*n* = 13) with a single *E. coli* strain were resistant to two or more antibiotics (Table 1). Patterns of antibiotic resistance were similar for *E. coli* isolates in CA-UTI, with and without considering polymicrobial infection (Supplementary Materials Figure S2).

### 3.3. Outcomes and Factors Associated with E. coli Resistance to Third Generation Cephalosporin

Patients with 3GCs-resistant *E. coli* CA-UTIs were more often boys (60.0% vs. 56.36%), individuals with a complex chronic disease (7.06% vs. 3.63%), and those recently taking antibiotics (18% vs. 11.65%) in comparison to patients in the 3GCs-susceptible group (Table 2). Details about the chronic disease involved, such as renal disease and progressive conditions, were more commonly found in the 3GCs-resistant group in comparison to the sensitive group, as presented in Supplementary Materials Table S4. Logistic regression analysis for 3GCs resistance found that complex chronic disease (aOR 2.04, 95%CI 1.47–2.83, *p* < 0.0001) and prior use of antibiotic treatment (one regimen vs. none: aOR 1.49, 95% CI (1.15–1.94), *p* = 0.0031) were correlated with a significant increased risk of acquiring *E. coli* CA-UTIs in the community. In addition, an increase in the number of prior antibiotic regimens (≥2 regimens vs. none: aOR 1.53, 95% CI (1.16–2.02), *p* = 0.0024) was associated with an increase in resistance to 3GCs (Table 3).

The in-hospital mortality rate after *E. coli* CA-UTIs was 0.36% and differed insignificantly between sensitive and resistant groups. Children with 3GCs-resistant *E. coli* CA-UTIs were more likely to have a longer length index hospitalization than those who were sensitive to 3GCs (mean 8.09 (±4.59) vs. 6.78 (±3.53) days, *p* < 0.0001) (Table 1). Among patients admitted for *E. coli* CA-UTIs, 1.44% (*n* = 106) had a discharge diagnosis of *E. coli* bloodstreams infections.

## 4. Discussion

From 2004 to 2018 in Taiwan, the trend of CA-UTIs caused by *E. coli* in this large multicenter hospitalized children cohort was evaluated. The trend observed for the incidence of UTIs (community- and healthcare-associated) caused by the *E. coli* pathogen was constant, but 3GC-resistant *E. coli* was found to significantly increase in community-associated UTIs over the duration of the study. An increase in resistance to ciprofloxacin, cefazolin, and gentamicin also occurred in children with *E. coli* isolated CA-UTIs.

In the literature, there are few longitudinal data studies of *E. coli* in pediatric patients with CA-UTIs. Previous reviews have suggested that *E. coli* accounts for 80–90% of community-onset UTIs in children [2,3]. The present study supports *E. coli* as the most common uropathogen causing UTIs among the participants, and it showed a steady trend over the 15-year period (ranging from 73.14 to 83.18% per year), which prior studies have not addressed.

The study results provide informative comparisons with urine microbiological results of confirmed UTIs in an adult population study in Oxfordshire, UK [12]. From 1998 to 2016, the overall incidence (first and recurrent) of *E. coli* in community-acquired UTIs appeared stable (IRR, 0.99; 95% CI, 0.98–1.00, *p* = 0.12) in the adult population [12]. Despite the use of different criteria to establish the incidence of community UTIs in the adult population, the steady trend observed is similar to that of our present child cohort (IRR, 1.01, 95% CI, 0.99–1.02, *p* = 0.3855). Notably, the frequencies of the common uropathogens involved were not different between adult and children, nor between geographic regions [27].

The incidence of ESBL-producing *E. coli* in community UTI has been increasing since 2001 [8], but the reported rates have varied across geographical regions, study settings, and the time at which the survey was conducted. In a global study for Monitoring Antimicrobial Resistance Trends from 2009 to 2011 (both children and adults), ESBL-producing UTI prevalence was high, and it increased over time in Asia (>40% in 2011), the Middle East, and the Latin American regions, while the incidence of resistance was reduced by <10% in North America and South Pacific regions [27].

A single center study performed in Turkey from 2008 to 2009 revealed that 41.4% of *E. coli* UTIs were ESBL producing [14]. In Spain, 9.2% of children aged 14 years or younger with community-onset *E. coli* UTI were ESBL-producing *E. coli* from 2015 to 2016 [20]. Another single-center study in the United States revealed the prevalence of ESBL-producing *E. coli* isolates in community UTIs ranged from 7 to 15% of all UTIs found in hospitalized children during a five-year period (2012–2016) [16]. Taken together, these finding suggest that the incidence of 3CGs-resistant *E. coli* (proxy of ESBL-*E. coli*) CA-UTI found in the present study was similar to North American and some European regions, during the same period of time (2008–2011: 1.86 to7.424% per year, and 2012–2016: 11.09 to 15.64%).

Ciprofloxacin is another common empirical therapy recommended for *E. coli* UTIs, and a second- or third-line therapy for complicated UTIs in children [22]. The present study found that the incidence of ciprofloxacin-resistant *E. coli* rapidly increased by over 20% since 2017 (2004–2011:6.85 to 9.74%; 2012–2018:11.09 to 28.26% per year). The ciprofloxacin-resistance trend in the present study and the 27% pooled resistance rate based on 47 studies worldwide (1998–2012) [28] reinforces the need to investigate the potential causes of antibiotic resistance in these geographical regions.

Unlike SMT-resistant *E. coli*, which was sustained over time, cefazolin- and gentamicin-resistant *E. coli* was found to increase slowly over the study period. The study results reveal that the increase in cefazolin (2013–2018: 33.28–41.68% per year) and gentamicin (2013–2018:21.88 to 31.66% per year) resistance is a matter of concern in the empirical antibiotic treatment of community UTIs in children. Alternative courses of treatment, including short-course antimicrobial therapy and empirical use of intravenous amikacin have been suggested [17,29]. In our study, almost all gentamicin-resistant *E. coli* strains were amikacin-sensitive, supporting the use of this antibiotic as an alternative for patients at risk of gentamicin-resistance.

Other important findings include the independent risk of 3GC-resistant *E. coli* in children with complex chronic diseases and the use of antibiotic treatments within three months prior to hospitalization for UTI. In hospitalized children, medical complexity accounts for a large proportion of hospital admissions, admission to an intensive care unit (ICU), and mortality [30,31]. Using PMCA, our study is the first to evaluate whether the complexity of chronic disease is related to 3GC-resistant *E. coli* in children with UTIs. Additionally, the study results support the notion that some children are at high risk of developing an antibiotic-resistance *E. coli* UTI, such as those having previous antibiotic exposure [5,11,32], spina bifida [33], and congenital abnormalities of the kidney and urinary tract [34]. However, congenital anomalies of the kidney and urinary tract (CAKUT) were ascertained by using diagnostic codes within one year prior to the index UTI hospitalization, and we found that CAKUT is not prevalent in this study cohort and not associated with an increased risk of 3GCs-resistant *E. coli* in childhood community UTI.

The present study shows a longer hospital stay among children with 3GCs-resistant *E. coli* UTI, which is supported by previous studies conducted in Taiwan and other geographic regions [9,11]. In-hospital mortality revealed no significant differences between 3GCs-resistant and -susceptible *E. coli* groups, which is possibly due to the fact that CA-UTIs were thought to represent a lower level of illness severity [35].

This study has several strengths. It is a longitudinal analysis, using a large multisite cohort to investigate trend changes over a 15-year period in children with 3GC-resistant UTIs in Taiwan. The American Academy of Pediatrics’ 2011 definition of a childhood UTI combines a clinically significant number of uropathogen (i.e., ≥10^5^ CFU/mL) and a hospital discharge diagnosis to minimize misclassification bias. Five common antibiotics for uncomplicated CA-UTIs were assessed for *E. coli* susceptibility, and children who are at risk for 3GC-resistant *E. coli* UTI were examined in this study, to guide future recommendations for empirical antibiotic treatment regimens.

The identification of risk factors and an adjustment of the local empirical treatment protocol, according to epidemiological surveillance data, are urgently warranted for children with CA-UTIs. B A narrow-spectrum empirical therapy (e.g., cefazolin, gentamicin, and amikacin) is recommended for patients with a suspected UTI, at the time of initial evaluation [22,23]. If broad-spectrum antibiotic empirical therapy were to be considered for severe symptoms and/or potential risk factors of 3GC-resistant uropathogens, it would have implications for antimicrobial stewardship to reduce the risk of inadequate antimicrobial treatment. A predictive algorithm (UTICalc calculator) that incorporates clinical and laboratory results has been proposed to assist clinicians in identifying UTIs in febrile children younger than two years [36]. The development of a predictive model, by evaluating the risk factors for antibiotic resistance to *E. coli* may be useful to assist in providing empirical antibiotic therapy recommendations.

There are several limitations in our study. Although the CGRD covered youths in a broad geographical region of Taiwan, there are numerous challenges related to the use of real-world data derived from a routine-care setting. First, important and clinically relevant data may be missing or not structured appropriately for the analysis. For instance, urine culture remains the gold standard for diagnosing UTIs. Although bacteria and antibiotic susceptibility are usually evident in properly plated urine specimens by 72 h post-collection, the record of urine sample collection time may be missing or late. In the present study, the date of validated culture results was used as a reference to define a community- or healthcare-associated UTI, where the time duration between the validated result and hospital admission (i.e., <4 or ≥4 days) was used to determine the origin of the UTI. Sensitivity analysis was conducted by using a cutoff time of three days, and similar results were obtained for the trend in the origin of the UTIs observed. Urine specimens collected in outpatient setting among children who did not receive further inpatient care were not assessed in the present study, which may lead to underestimated incidence of community UTIs. Further research should be focused on the frequency and results of urine culture testing in outpatient visits, to validate the present study results. Although possible small numbers of healthcare-associated UTIs developed within three days of hospitalization could be misclassified as CA-UTIs in the hospitalized children cohort, which might have less of an impact on CA-UTIs incidence as healthcare-associated UTIs remained steady and low incidence in the present and European studies [37]. Second, most urine samples were collected by using a urine bag in the study setting. Although culturing specimens from the bag reach a sensitivity of nearly 100%, the specificity is only within the range of 14 to 84% [38]. In addition, the study results might not able to extrapolate the exact resistant rate of ESBL-producing *E. coli* to 3GC in childhood UTIs because there are other resistance mechanisms that can confer resistance to the same antibiotics, such as AmpC beta-lactamases, carbapenemases, and, to a lesser extent, porin changes. Third, the therapeutic protocol for UTIs and antibiotic prophylaxis in the study setting may not be generalizable to other practice settings or child populations.

## 5. Conclusions

In conclusion, this study found no change in the trend of *E. coli* isolates in CA-UTIs, in Taiwanese youths, over a 15-year study period. However, the antibiotic resistance of *E. coli* UTIs in the community setting continues to increase, especially for 3GCs and ciprofloxacin. Inpatient care and duration of hospital stays was found to be higher in patients with 3GCs-resistance than those with 3GCs-susceptible *E. coli* CA-UTIs. Strategies are needed to evaluate potential risk factors associated with community-onset 3GCs- and ciprofloxacin-resistant *E. coli* UTIs in Taiwan, and globally. Further research aimed at developing predictive tools to identify risk factors associated with resistant pathogens in children with UTIs will provide a significant clinical improvement in this area.

## Figures and Tables

**Table 1:** Healthcare service use and patient outcomes among children with community-associated *E. coli* urinary tract infection.

Heading	Overall *	3GCs-Resistant (*n* = 822)		3GCs-Susceptible (*n* = 6556)		*p*-Value #
	N	n	(%)	n	(%)	
In-hospital death	27	6	(0.73)	21	(0.32)	0.1133
Length of stay (day),						<0.0001
Mean (SD)	7378	8.09	(4.59)	6.78	(3.53)	
Median (IQR)		7.00	(5.00–10.00)	6.00	(5.00–8.00)	
Discharge diagnosis with bacteremia	311	45	(5.47)	266	(4.06)	0.0566
*E. coli*	106	13	(1.58)	93	(1.42)	0.7113

Abbreviations: 3GCs, third-generation cephalosporins; SD, standard deviation; IQR, interquartile range (25th–75th percentile) * Community-associated *E. coli* urinary tract infection indicated patients with single-isolated *E. Coli* uropathogen. # Differences between groups for categorical variables were tested by using Chi square tests and independent Sample *t*-tests for numeric variables.

**Table 2:** Characteristics of children with community-associated *E. coli* urinary tract infection, 2004–2018.

Heading	Overall *	3GCs-Resistant (*n* = 822)		3GCs-Susceptible (*n* = 6556)		*p*-Value #
	n	n	(%)	n	(%)	
Age at admission						0.4516
≤28 days	379	39	(4.74)	340	(5.19)	
29 days to < 1 year	5534	634	(77.13)	4900	(74.74)	
1 to 2 years	808	86	(10.46)	722	(11.01)	
3 to 17 years	657	63	(7.66)	594	(9.06)	
Sex						0.0415
Boys	4189	494	(60.10)	3695	(56.36)	
Girls	3189	328	(39.90)	2861	(43.64)	
**Recent medical visits ≤ 3 months prior, visit**						
Outpatient						0.4501
None	6410	721	(87.71)	5689	(86.78)	
1–3	722	71	(8.64)	651	(9.93)	
≥4	246	30	(3.65)	216	(3.29)	
Emergency department						0.2825
None	6911	764	(92.94)	6147	(93.76)	
1	365	49	(5.96)	316	(4.82)	
≥2	102	9	(1.09)	93	(1.42)	
Hospitalization						0.0834
None	7070	784	(95.38)	6286	(95.88)	
1	279	31	(3.77)	248	(3.78)	
≥2	29	7	(0.85)	22	(0.34)	
**PMCA, CD**						<0.0001
Without CD	6640	724	(88.08)	5916	(90.24)	
Non-complex CD	442	40	(4.87)	402	(6.13)	
Complex CD	296	58	(7.06)	238	(3.63)	
**Recent antibiotic treatment ≤ 3 months prior**						
**Sum of regimen**						<0.0001
None	6427	674	(82.00)	5753	(87.75)	
1	515	79	(9.61)	436	(6.65)	
≥2	436	69	(8.39)	367	(5.60)	
Amoxicillin/clavulanic acid	286	49	(5.96)	237	(3.62)	0.0010
Amikacin	11	6	(0.73)	5	(0.08)	<0.0001
Ciprofloxacin	9	5	(0.61)	4	(0.06)	<0.0001
Ceftriaxone	38	9	(1.09)	29	(0.44)	0.0137
Cefazolin	37	10	(1.22)	27	(0.41)	0.0021
Ertapenem	412	63	(7.66)	349	(5.32)	0.0059
Gentamicin	550	75	(9.12)	475	(7.25)	0.0532
Levofloxacin	1	0	(0.00)	1	(0.02)	--
Sulfamethoxazole–Trimethoprim	133	36	(4.38)	97	(1.48)	<0.0001
Piperacillin/Tazobactam	2	1	(0.12)	1	(0.02)	--

Abbreviations: 3GCs, third-generation cephalosporins; PMCA, Pediatric Medical Complexity Algorithm; CD, chronic disease. * Community-associated *E. coli* urinary tract infection indicated patients with single-isolated *E. Coli* uropathogen. # Differences between groups for categorical variables were tested using Chi square tests or Fischer’s exact tests (sample size < 5).

**Table 3:** Factors associated with third-generation cephalosporin resistance of *E. coli* in the community-associated UTI cohort.

Heading	aOR	95%CI		*p*-Value
Age at index hospitalization				
≤28 days	1			
29 days to < 1 year	1.08	(0.77	1.52)	0.6589
1 to 2 years	0.98	(0.65	1.47)	0.9059
3 to 17 years	0.80	(0.51	1.24)	0.3075
Boy (vs. girl)	1.13	(0.96	1.32)	0.1337
**Healthcare service use ≤3 months prior, visit**				
Outpatient setting				
None	1			
1–3	0.80	(0.60	1.08)	0.1449
≥4	0.70	(0.43	1.13)	0.1434
Emergency department				
None	1			
1	1.34	(0.95	1.88)	0.0910
≥2	0.77	(0.37	1.59)	0.4785
Hospitalization				
None	1			
1	0.98	(0.63	1.53)	0.9414
≥2	2.15	(0.83	5.58)	0.1176
PMCA, CD				
Non-Chronic	1			
Non-complex Chronic	0.83	(0.59	1.18)	0.3005
Complex Chronic	2.04	(1.47	2.83)	<0.0001
**Sum of antibiotic regimen use ≤ 3 months prior**				
None	1			
1	1.49	(1.15	1.94)	0.0031
≥2	1.53	(1.16	2.02)	0.0024

Abbreviations: aOR, adjusted odds ratio; CI, confidence interval; PMCA, Pediatric Medical Complexity Algorithm; CD, chronic disease.

## Supplementary Materials

The following are available online at https://www.mdpi.com/2079-6382/9/8/501/s1. Figure S1: Trends of overall *E. coli* (single and polymicrobial) in community-associated UTIs among children aged < 18 years, 2004–2018 (IRR: 1.01 95% CI (0.99–1.02), *p* = 0.3855). Figure S2: Trends of antibiotic resistance of overall *E. coli* (single and polymicrobial) in community-associated UTIs among children aged < 18 years, 2004–2018. Table S1: Codes and definitions for the study cohort. Table S2: Annual hospital Admissions and *E. coli* single isolated uropathogen UTIs. Table S3: Characteristics of incident *E. coli* UTI cohort between community- and healthcare-associated subgroups (*n* = 7696). Table S4: Comorbid condition of the *E. coli* community-associated UTI cohort.
