# Supplementary material for: Trends in Antimicrobial Susceptibility of Escherichia coli Isolates in a Taiwanese Child Cohort with Urinary Tract Infections between 2004 and 2018
Source: Antibiotics (Basel). 2020 Aug 10;9(8):501. doi: 10.3390/antibiotics9080501 (PMC7460002; doi:10.3390/antibiotics9080501)
Supplement: Supplementary file 1 [file antibiotics-09-00501-s001.pdf]

Supplementary Data

Table S1. Codes and definitions for the study cohort.

Variable	Codes	Definition
Urinary tract infection (UTI)	ICD-9-CM: 590.X, 595.0, 599.0 ICD-10-CM: N39.0	Diagnosis at hospital discharge
Pediatric Medical Complexity Algorithm (PMCA)	ICD9/10-CM	ICD codes for a disease diagnosis in outpatient/ED (≥ 2 codes at different date) or inpatient setting (≥1 codes), ≤ 3 months prior to the index hospitalization
Bacteremia (*E. coli*)	ICD-9-CM: 03842 or ICD-10-CM: A4151	Diagnosis at hospital discharge
Antibiotic regiment, by ATC codes		ATC codes for recent antibiotic treatments in outpatient setting, ≤ 3 months prior to the index hospitalization
Amoxicillin/clavulanic acid	J01CA04, J01CR02	Outpatient setting
Amikacin	J01GB06	Outpatient setting
Ciprofloxacin	J01MA02	Outpatient setting
Ceftriaxone	J01DD04	Outpatient setting
Cefazolin	J01DB04	Outpatient setting
Ertapenem	J01DH03	Outpatient setting
Gentamicin	J01GB03	Outpatient setting
Levofloxacin	J01MA12	Outpatient setting
Sulfamethoxazole–Trimethoprim	J01EE01	Outpatient setting
Piperacillin/Tazobactam	J01CR05, J01CA12	Outpatient setting

Abbreviations: ICD-9/10-CM, International Classification of Diseases, Ninth and Tenth Revisions, Clinical Modification; PMCA, Pediatric Medical Complexity Algorithm; ATC, anatomical therapeutic chemical classification system.

Table S2. Annual hospital admissions and *E. coli* single isolated uropathogen UTIs.

Year	Total Hospital Admissions, n	Person with Incident UTI Diagnosis, n	Person with Urine Cultures, n	Incident UTI Case with any Uropathogen >10^5 CFU/mL, n	Community with Single *E. coli* Isolated, n	Healthcare with Single *E. coli* Isolated, n
2004	20,233	1923	1522	309	226	18
2005	50,072	1936	1678	411	298	27
2006	49,076	1978	1758	563	429	26
2007	48,306	2094	1903	626	465	26
2008	49,465	2107	1897	655	483	27
2009	47,680	2039	1827	624	487	13
2010	55,247	1567	1381	595	464	21
2011	47,574	1723	1540	572	437	21
2012	45,558	1866	1021	541	450	17
2013	43,241	2226	2010	860	681	37
2014	45,695	2339	2151	978	745	51
2015	44,980	2188	1987	923	758	33
2016	46,739	1821	1613	719	573	22
2017	44,503	1948	1707	735	590	16
2018	42,681	1727	1509	634	506	22
Sum	711,050	29482	25504	9745	7592	377
IRR					1.01 (0.99–1.02), p = 0.2870	0.96 (0.90–1.02), p = 0.1724

Abbreviations: UTI, urinary tract infection; IRR, incidence rate ratio; CFU, colony-forming unit. Overall IRR for single-isolated *E. coli* UTI was 1.00 (0.99–1.02), p = 0.4612 (community- and healthcare-associated UTI) during the study period.

Table S3. Characteristics of incident *E. coli* UTI cohort between community- and healthcare-associated subgroups (n = 7696).

Variable	Overall*	Community (n = 7592)		Healthcare (n = 377)		p-value[a]
	n	n	(%)	n	(%)	
**Age at admission, year, mean (SD)**	7969	1.22	(2.60)	2.42	(4.57)	<0.0001
**Age group**						<0.0001
≤28 days	525	407	(5.36)	118	(31.30)	
< 1 year	5814	5682	(74.84)	132	(35.01)	
1–2 years	890	834	(10.99)	56	(14.85)	
3–17 years	740	669	(8.81)	71	(18.83)	
**Sex**						0.0550
Boy	4503	4308	(56.74)	195	(51.72)	
Girl	3466	3284	(43.26)	182	(48.28)	
**Recent medical visits ≤ 3 months prior, visit**						
Outpatient setting						<0.0001
None	6902	6612	(87.09)	290	(76.92)	
1–3	784	732	(9.64)	52	(13.79)	
≥4	283	248	(3.27)	35	(9.28)	
Emergency department						<0.0001
None	7434	7122	(93.81)	312	(82.76)	
1	410	367	(4.83)	43	(11.41)	
≥2	125	103	(1.36)	22	(5.84)	
Hospitalization						<0.0001
None	7607	7279	(95.88)	328	(87.00)	
1	321	284	(3.74)	37	(9.81)	
≥2	41	29	(0.38)	12	(3.18)	
**Baseline CKD**						0.9301
No CKD	7852	7479	(98.51)	373	(98.94)	
Non-CAKUT	16	15	(0.20)	1	(0.27)	
CAKUT	40	39	(0.51)	1	(0.27)	
Risk group#	61	59	(0.78)	2	(0.53)	
**PMCA, CD**						
**More conservative algorithm**						<0.0001
Without CD	7141	6835	(90.03)	306	(81.17)	
Non-complex CD	475	451	(5.94)	24	(6.37)	

Complex CD	353	306	(4.03)	47	(12.47)	
**Sum of antibiotic regiments ≤ 3 months prior**						0.2146
None	6942	6619	(87.18)	323	(85.68)	
1	567	532	(7.01)	35	(9.28)	
≥2	460	441	(5.81)	19	(5.04)	
Amoxicillin/clavulanic acid	314	289	(3.81)	25	(6.63)	0.0059
Amikacin	15	11	(0.14)	4	(1.06)	0.0001
Ciprofloxacin	11	9	(0.12)	2	(0.53)	0.0355
Cefazolin	44	41	(0.54)	3	(0.80)	0.5131
Gentamicin	40	37	(0.49)	3	(0.80)	0.4082
Levofloxacin	434	417	(5.49)	17	(4.51)	0.4115
Sulfamethoxazole–Trimethoprim	584	561	(7.39)	23	(6.10)	0.3487
Piperacillin/Tazobactam	1	1	(0.01)	0	(0.00)	0.8236

CKD: chronic kidney disease; CAKUT: congenital anomalies of kidney and urinary tract; PMCA: Pediatric Medical Complexity Algorithm; CD: chronic disease. *Community- and healthcare-associated *E coli* urinary tract infection indicated patients with single-isolated *E Coli* uropathogen. #risk group: hematuria, hydronephrosis, acute kidney injury. [a] Differences between groups for categorical variables were tested using Chi square tests or Fischer's exact tests (sample size < 5), and independent Sample t tests for numeric variables.

Table S4. Comorbid condition of the *E. coli* community-associated UTI cohort.

Comorbidity	Overall	3GCs-Resistant (*n* = 822)		3GCs-Susceptible (*n* = 6556)		*p*-value[a]
	n	n	(%)	n	(%)	
**CKD diagnosis, prior***						0.2172
No CKD	7269	804	(97.81)	6465	(98.61)	
Non-CAKUT	14	2	(0.24)	12	(0.18)	
CAKUT#	37	6	(0.73)	31	(0.48)	
Risk group$	58	10	(1.22)	48	(0.73)	
**PMCA, organ system***						
Cardiac	137	31	(3.77)	106	(1.62)	<0.0001
Craniofacial	22	3	(0.36)	19	(0.29)	0.7095
Dermatological	1	0	(0.00)	1	(0.02)	
Endocrinological	26	2	(0.24)	24	(0.37)	0.5755
Gastrointestinal	89	13	(1.58)	76	(1.16)	0.2958
Genetic	38	3	(0.36)	35	(0.53)	0.5237
Genitourinary	131	17	(2.07)	114	(1.74)	0.5004
Hematological	14	4	(0.49)	10	(0.15)	0.0380
Immunological	17	2	(0.24)	15	(0.23)	0.9348
Malignancy	17	6	(0.73)	11	(0.17)	0.0015
Mental health	78	8	(0.97)	70	(1.07)	0.8028
Metabolic	31	3	(0.36)	28	(0.43)	0.7952
Musculoskeletal	28	7	(0.85)	21	(0.32)	0.0195
Neurological	182	28	(3.41)	154	(2.35)	0.0654
Ophthalmological	33	6	(0.73)	27	(0.41)	0.1976
Otologic	19	2	(0.24)	17	(0.26)	0.9320
Pulmonary/Respiratory	153	10	(1.22)	143	(2.18)	0.0673
Renal	82	17	(2.07)	65	(0.99)	0.0055
Progressive	155	31	(3.77)	124	(1.89)	0.0004

Abbreviations: CKD, chronic kidney disease; CAKUT, congenital anomalies of kidney and urinary tract; PMCA, Pediatric Medical Complexity Algorithm; CD, chronic disease. *CKD and PMCA were retrieved within three months prior to the community-acquired *E. coli* UTI index hospitalization. #CAKUT group included patients (*n* = 2) ever diagnosed CAKUT and other etiology of CKD (non-CAKUT). $Risk group: hematuria, hydronephrosis, and acute kidney injury. [a] Differences between groups for categorical variables were tested using Chi square tests

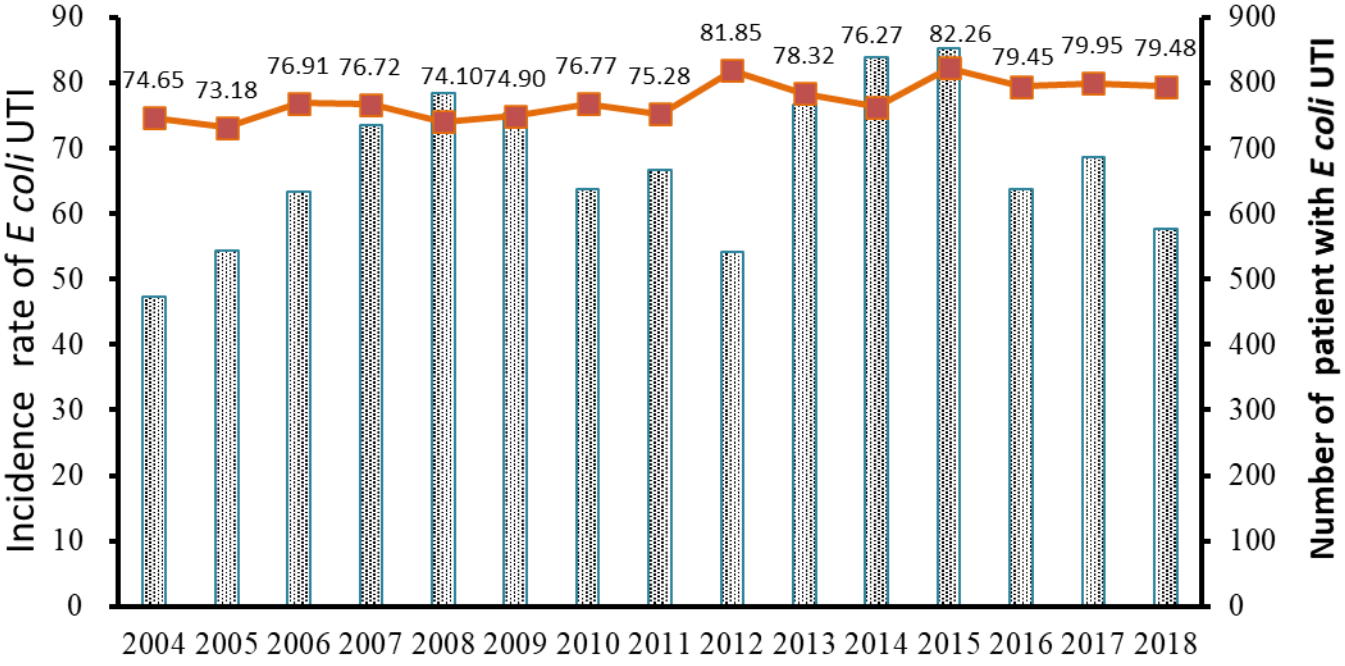

Figure S1. Trends of overall *E. coli* in community-associated UTI (single and polymicrobial) among children aged < 18 years, 2004–2018 (IRR: 1.01 95%CI (0.99–1.02), $p = 0.3855$).

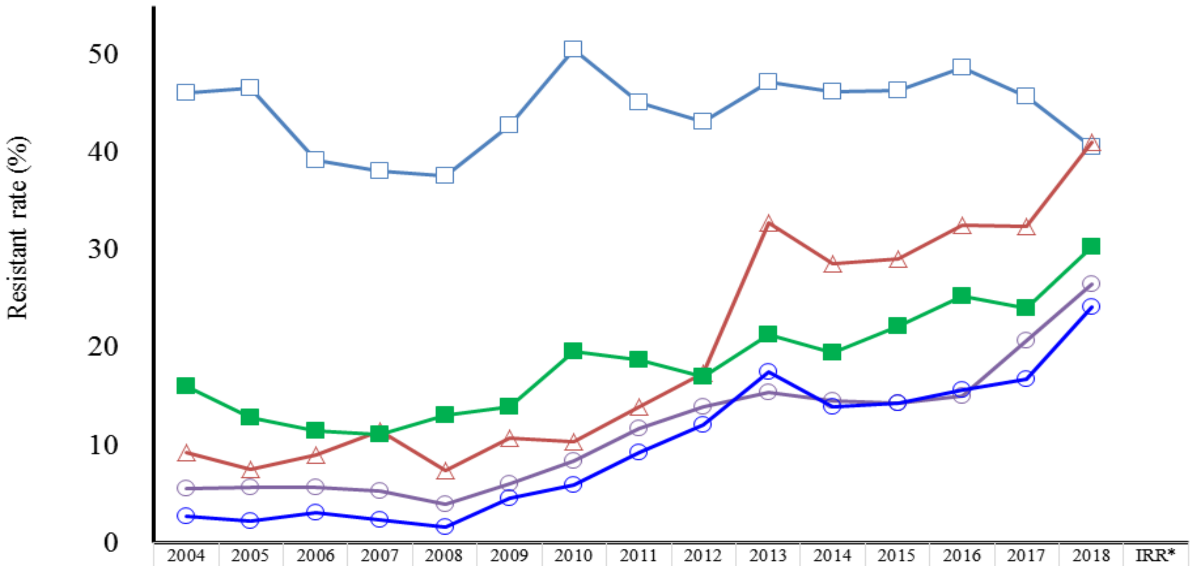

	2004	2005	2006	2007	2008	2009	2010	2011	2012	2013	2014	2015	2016	2017	2018	IRR*
SMT	46.09	46.67	39.21	38.08	37.56	42.80	50.56	45.16	43.13	47.17	46.26	46.41	48.74	45.78	40.53	1.01
Cefazolin	9.25	7.51	9.00	11.51	7.44	10.66	10.33	13.98	17.33	32.75	28.55	29.11	32.49	32.44	41.05	1.14
Gentamicin	16.07	12.75	11.41	11.05	13.01	13.95	19.55	18.74	16.95	21.29	19.45	22.17	25.24	24.00	30.35	1.06
Ciprofloxacin	5.48	5.65	5.71	5.32	3.96	6.09	8.43	11.67	13.94	15.36	14.59	14.25	15.01	20.65	26.49	1.13
3GC	2.75	2.22	3.02	2.33	1.54	4.57	5.88	9.22	12.05	17.52	13.97	14.25	15.62	16.74	24.21	1.18

Figure S2. Trends of antibiotic resistance of overall *E. coli* community-associated UTI (single and polymicrobial) among children aged<18 years, 2004-2018. SMT: Sufalmethoxazole-trimethoprim; 3GCs: Third generation cephalosporins.

IRR*: incidence rate ratio, all p values <0.0001, except SMT (p = 0.5647). The mean IRR was derived from a Poisson regression analysis based on the observed rate in each calendar year. The denominator was the patients with E coli isolated UTI having the particular antibiotic susceptibility results across the study period. Resistance among children aged < 18 years, 2004–2018.
